# Development of a Four-mRNA Expression-Based Prognostic Signature for Cutaneous Melanoma

**DOI:** 10.3389/fgene.2021.680617

**Published:** 2021-07-15

**Authors:** Haiya Bai, Youliang Wang, Huimin Liu, Junyang Lu

**Affiliations:** ^1^Department of Female Plastic Surgery, Gansu Provincial Maternity and Child-Care Hospital, Lanzhou, China; ^2^Department of Pediatric Surgery, Gansu Provincial Maternity and Child-Care Hospital, Lanzhou, China

**Keywords:** cutaneous melanoma, prognostic signature, random survival forest, MRNA expression data, machine learning

## Abstract

We aim to find a biomarker that can effectively predict the prognosis of patients with cutaneous melanoma (CM). The RNA sequencing data of CM was downloaded from The Cancer Genome Atlas (TCGA) database and randomly divided into training group and test group. Survival statistical analysis and machine-learning approaches were performed on the RNA sequencing data of CM to develop a prognostic signature. Using univariable Cox proportional hazards regression, random survival forest algorithm, and receiver operating characteristic (ROC) in the training group, the four-mRNA signature including CD276, UQCRFS1, HAPLN3, and PIP4P1 was screened out. The four-mRNA signature could divide patients into low-risk and high-risk groups with different survival outcomes (log-rank *p* < 0.001). The predictive efficacy of the four-mRNA signature was confirmed in the test group, the whole TCGA group, and the independent GSE65904 (log-rank *p* < 0.05). The independence of the four-mRNA signature in prognostic prediction was demonstrated by multivariate Cox analysis. ROC and timeROC analyses showed that the efficiency of the signature in survival prediction was better than other clinical variables such as melanoma Clark level and tumor stage. This study highlights that the four-mRNA model could be used as a prognostic signature for CM patients with potential clinical application value.

## Introduction

Cutaneous melanoma (CM) is a highly aggressive and heterogeneous skin malignancy. In recent years, the morbidity and mortality of CM have increased significantly (Dimitriou et al., 2018), with approximately 232,100 new cases and 55,500 death each year (Schadendorf et al., 2018). Although CM is usually detected in T1 stage and the corresponding patients’ 5-year survival exceeds 90%, the rapid progression of melanoma and the failure to detect thin melanoma in time lead to the progression and metastasis, which worsen the prognosis of CM patients [the 5-year survival rate is reduced to 62.9% (Dinnes et al., 2018) for regional lymph nodes spread and 19% (Miller et al., 2019) for distant metastasis]. Classic prognostic factors, including age and American Joint Committee on Cancer (AJCC) stage, have been proven to be effective indicators for melanoma (Kretschmer et al., 2011; Shields et al., 2012, 2015). Melanoma-specific indicators, including Clark level and Breslow thickness (Morton et al., 1993; Panda et al., 2018), are also used to assess the survival of CM patients. However, these clinicopathological indicators cannot reflect the molecular heterogeneity of melanoma (Palmieri et al., 2018), nor can they accurately predict the clinical outcome. Thus, novel prognostic biomarkers are extremely necessary for CM patients.

The development of sequencing technology and bioinformatics tools has promoted the discovery of new tumor biomarkers and the study of tumor molecular mechanisms. Based on the analysis of public messenger RNA (mRNA) expression data, studies have shown that gene signature could be prognostic marker for different types of tumors. For instance, a nine-gene signature can reliably predict the overall survival of patients with pancreatic cancer (Wu et al., 2019). An eight-gene signature showed a robust prognostic performance in early-stage non-small cell lung cancer (He et al., 2019). A 15-gene signature has been found to divide colon cancer patients into two groups with different prognosis (Xu et al., 2017). The prognostic two-gene signature has presented good predictive ability in the prognosis of GBM patients (Pan et al., 2020). For melanoma, Wang et al. (2020) identified that an eight-gene signature could independently predict the poor clinical outcome of melanoma patients.

It is well known that signatures with fewer genes have better clinical significance. In this study, through mining the mRNA expression profile and clinical information of 385 CM patients by statistical and machine-learning analyses, we aim to evaluate the prognostic significance of all expressed mRNAs and construct an effective prognostic signature for CM patients.

## Materials and Methods

### Expression Profile of CM Patients

The clinical parameters and mRNA expression data of CM in The Cancer Genome Atlas (TCGA) database^1^ were from UCSC Xena^2^. The CM cases with clinical survival follow-up information were selected to establish a prognostic model, which were randomly divided into training and test groups. The independent validation set (GSE65904) was obtained from Gene Expression Omnibus (GEO) database. The clinical details of CM patients including age; gender; pathological T, N, and M stage; and tumor stage are displayed and summarized in Supplementary Table 1. We discarded genes whose expression values were missing in more than 20% of CM samples. All expression values were log2 transformed.

### The Process of Developing Prognostic Models Through Statistics and Machine-Learning Methods

Univariable Cox analysis was applied to seek mRNAs significantly correlated with CM patients’ OS in the training group. Then, random survival forest (RSFVH, a machine learning approach) was performed. In RSFVH, an iteration procedure was implemented to reduce the node set in which the one-third least important mRNAs were discarded at each iteration step (Li et al., 2014). As a result, we obtained a set of prognosis-related mRNAs that contained a relatively small number. Subsequently, we constructed prognostic combination models as the following formula:

$$\text{Risk score (RS)}=\Sigma_{i=1}^{N}\,\left({\text{coefficient}}_{i}^{*}\,\text{mRNA}\,{\text{Exp}}_{i}\right)$$

where N is the number of prognosis-related mRNAs, **mRNA*Exp*** is the expression value of prognosis-related mRNA, and the coefficient of prognosis-related mRNA is derived from Cox regression. The prognostic RS model was selected for its largest area under the curve (AUC) value from all the combinations (Zhang et al., 2020).

### Statistical Analysis

Log rank test and Kaplan–Meier (KM) analysis were performed to analyze the difference in survival between the two groups separated by the median risk score. Chi-square test was performed to test the association of the selected signature with other clinical parameters. The predictive performance of the signature in survival was tested by receiver operating characteristic (ROC) and time-dependent ROC. The R program (**⁇**) performed the above analyses using R packages named pROC, randomForestSRC, and survival. The prognostic mRNAs were analyzed by Gene Ontology (GO) and Kyoto Encyclopedia of Genes and Genomes (KEGG) analysis through the Cluego plug-in of Cytoscape software (Bindea et al., 2009).

## Results

### Development of the Prognostic Four-mRNA Signature

From the TCGA database, we obtained 385 patients diagnosed with CM and their mRNA expression profiles including a total of 18,496 expressed mRNAs. After summarizing the clinical characteristics of CM patients, we found that the median age was 58 years (15–87 years), and a large proportion of patients were men, indicating that CM was more common in male adults. Additionally, we found that the median survival time of CM patients was 37.5 months, and only a small number of people were still alive, confirming the poor prognosis of CM (Supplementary Table 1).

**FIGURE 1:**
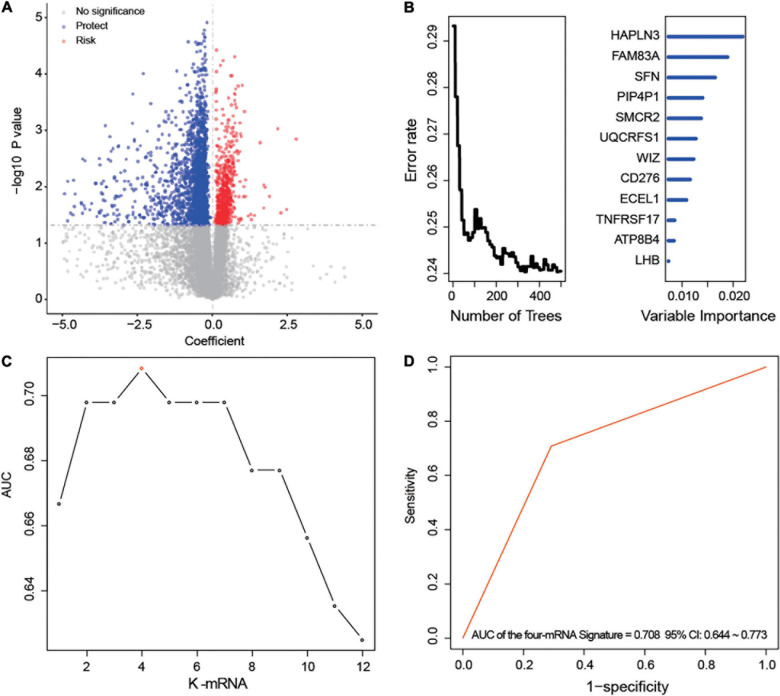
Development of the prognostic messenger RNA (mRNA) signature. **(A)** The survival-associated mRNAs obtained from Cox analysis are displayed on the volcano plot. **(B)** After random forest classification algorithm, the prognosis-associated mRNAs were decreased to 12. **(C,D)** The prognostic four-mRNA signature was selected because its area under the curve (AUC) was the largest (AUC = 0.708) among the 2^12^–1 = 4,095 signatures.

After univariable Cox regression, we discovered 3,058 mRNAs that were significantly related to survival (red and blue dots in Figure 1A, *p* < 0.05). Subsequently, we screened out 12 prognostic mRNAs based on the importance score of RSFVH analysis (Figure 1B). When these 12 prognostic mRNAs were incorporated into the risk prediction model in different combinations, we got a total of 2^12^–1 = 4,095 possible signatures. After performing ROC analysis on all 4,095 signatures, we found a four-mRNA signature with the largest AUC value (AUCsignature = 0.708; Figures 1C,D and Table 1). The prognostic four mRNAs from the signature were CD276, HAPLN3, PIP4P1, and UQCRFS1. The selected RS model formula was as follows: RS = (0.35 × CD276 expression level) + (0.75 × UQCRFS1 expression level) + (-0.41 × HAPLN3 expression level) + (-0.75 × PIP4P1 expression level).

**TABLE 1:** Survival analysis of the messenger RNAs (mRNAs) in the prognostic signature.

**geneID**	**HR**	**Right**	**Left**	**COX P**	**KM P**
CD276	1.42	1.07	1.88	0.01	0.03
HAPLN3	0.66	0.55	0.81	<0.001	<0.001
PIP4P1	0.47	0.30	0.74	<0.001	<0.001
UQCRFS1	2.13	1.43	3.16	<0.001	<0.001

### The Survival Prediction Performance and Validation of the Four-mRNA Signature

**FIGURE 2:**
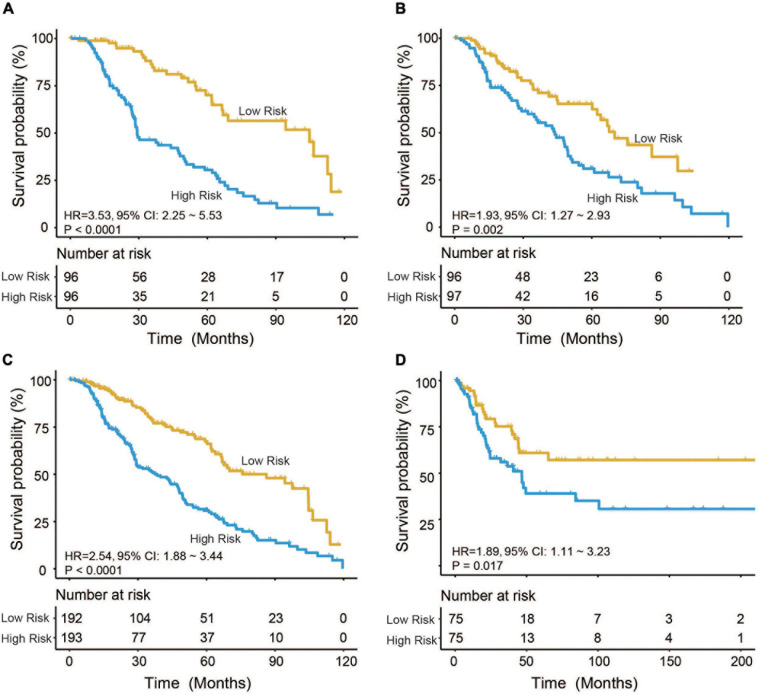
Cutaneous melanoma patients were divided by the four-messenger RNA (four-mRNA) signature into two risk groups with significantly different survival outcomes in the **(A)** training, **(B)** test, **(C)** entire The Cancer Genome Atlas (TCGA), and **(D)** GSE65904 datasets.

**FIGURE 3:**
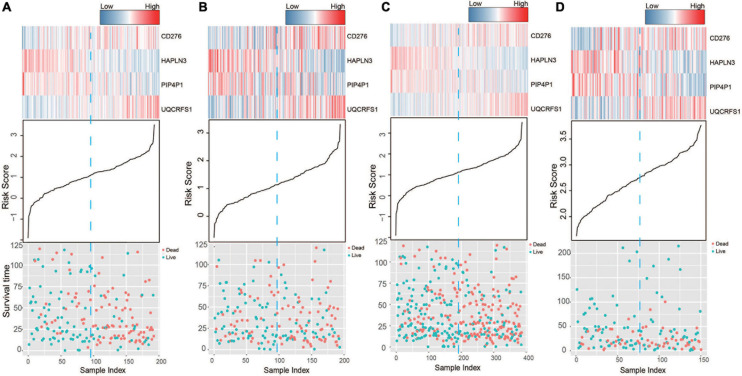
The risk score distribution, survival status, and messenger RNA (mRNA) expression patterns of cutaneous melanoma patients in the **(A)** training, **(B)** test, **(C)** entire The Cancer Genome Atlas (TCGA), and **(D)** GSE65904 datasets.

Based on the model constructed above, the risk scores of CM patients were calculated, and the median risk score was obtained as the cutoff. In the training dataset, the median RS divided patients into a high-risk group (*n* = 96) or a low-risk group (*n* = 96). Kaplan–Meier analysis showed that there were a significant difference in survival time between patients in the high- and the low-risk group (median survival time: 29.2 vs. 104.7 months, *p* < 0.001; Figure 2A). Then, Kaplan–Meier analysis was performed in the test (*n* = 193) and entire TCGA datasets (*n* = 385). The four-mRNA signature could distinguish the CM patients into two risk groups with different survival outcomes in the test group (median survival time: 43.8 vs. 70.0 months, *p* < 0.001; Figure 2B). The same performance for survival prediction was shown in the entire TCGA dataset (median survival time: 38.5 vs. 86.3 months, *p* < 0.001; Figure 2C). In addition, we also verified its survival prediction performance in an independent set (GSE65904, *n* = 150) from GEO database. The median RS value also classified patients from GSE65904 into high- or low-risk group significantly (*p* = 0.017, Figure 2D). Moreover, when the patient’s mRNA expression, survival time, and risk score were displayed on the same chart, we found that CM patients with higher risk mRNAs expression and higher risk scores had poorer survival outcomes in the training (Figure 3A), test (Figure 3B), entire TCGA datasets (Figure 3C), and GSE65904 (Figure 3D).

### Independent Prognostic Value of the Four-mRNA Signature

Chi-square test did not detect the relationship between the four-mRNA signature and other clinical variables (Table 2). Then, we tested the independence of the four-mRNA model by performing Cox regression analysis (including univariate and multivariable Cox, Table 3). Multivariable Cox regression confirmed the independence of four-mRNA signature in prognosis prediction in the training, test, entire TCGA, or GSE65904 datasets (HR training = 3.00, *p* < 0.001, *n* = 192; HR test = 1.72, *p* < 0.05, *n* = 193; HR entire = 2.00, *p* < 0.001, *n* = 385; HRGSE65904 = 2.25, *p* < 0.001, *n* = 150, Table 3).

**TABLE 2:** Association of the messenger RNA (mRNA) signature with clinical characteristics in cutaneous melanoma (CM) patients.

**Variables**	**Training group**		***p***	**Test group**		***p***	**TCGA group**		***p***
	Low risk*	High risk*		Low risk*	High risk*		Low risk*	High risk*	
Age (years)			0.89			0.27			0.57
≤ 58	48	46		38	47		86	93	
>58	48	50		58	50		106	100	
Gender			0.38			1.00			0.50
Female	43	36		33	33		76	69	
Male	53	60		63	64		116	124	
Radiotherapy			<0.001			0.13			<0.001
Unknown	25	50		29	41		54	91	
No	63	42		57	51		120	93	
Yes	8	4		10	5		18	9	
Pathological M			0.33			0.52			0.21
M0	84	89		82	86		166	175	
M1	6	5		5	6		11	11	
Pathological N			0.56			0.31			0.19
N0	48	44		42	37		90	81	
N1	14	19		19	16		33	35	
N2	11	9		12	12		23	21	
N3	11	16		10	18		21	34	
Pathological T			0.17			0.31			0.27
T0	5	1		6	7		11	8	
T1	12	9		8	1		20	10	
T2	16	17		12	14		28	31	
T3	16	25		17	17		33	42	
T4	30	37		35	41		65	78	
Tumor stage			0.31			0.87			0.38
Stage 0	0	1		1	0		1	1	
Stage I	20	14		11	9		31	23	
I/II Nos	2	0		1	0		3	0	
Stage II	29	31		29	31		58	62	
Stage III	32	42		41	44		73	86	
Stage IV	6	5		5	6		11	11	
Race demographic			0.22			0.03			0.51
Asian	3	3		2	4		5	7	
White	93	90		88	93		181	183	

*Low risk ≤ median of risk score, high risk > median of risk score.

**TABLE 3:** Cox regression analysis of the signature with the survival of cutaneous melanoma (CM).

		**Univariable analysis**				**Multivariable analysis**			
**Variables**		**HR**	**95% CI of HR**		***P***	**HR**	**95% CI of HR**		***p***
			**lower**	**upper**			**lower**	**upper**	
**The training group**									
Age	>58 vs. ≤58	1.47	0.98	2.22	0.07	1.24	0.77	2.01	0.38
Gender	Male vs. female	0.79	0.52	1.20	0.27	1.03	0.64	1.67	0.89
Tumor stage	III, IV vs. I, II	1.01	0.98	1.03	0.60	1.03	0.97	1.08	0.36
Melanoma Clark level	IV, V vs. I, II, III	1.87	1.16	3.02	0.01	1.40	1.01	1.94	0.04
Signature	High risk vs. low risk	3.53	2.25	5.53	< 0.001	3.00	1.77	5.06	< 0.001
**The test group**									
Age	>58 vs. ≤58	0.87	0.58	1.31	0.51	0.85	0.51	1.41	0.52
Gender	Male vs. female	1.01	0.65	1.58	0.96	0.75	0.43	1.31	0.31
Tumor stage	III, IV vs. I, II	0.99	0.97	1.02	0.56	0.98	0.94	1.01	0.24
Melanoma Clark level	IV, V vs. I, II, III	1.35	0.86	2.11	0.20	1.33	0.94	1.89	0.11
Signature	High risk vs. low risk	1.93	1.27	2.93	<0.001	1.72	1.02	2.90	0.04
**The TCGA dataset**									
Age	>58 vs. ≤58	1.13	0.85	1.50	0.40	0.90	0.50	1.64	0.74
Gender	Male vs. female	0.90	0.67	1.22	0.51	0.98	0.52	1.85	0.95
Tumor stage	III, IV vs. I, II	1.00	0.98	1.01	0.80	1.04	0.99	1.10	0.14
Melanoma Clark level	IV,V vs. I, II,III	1.55	1.13	2.13	0.01	1.88	1.19	2.96	0.01
Radiation therapy	Yes vs. no	0.74	0.35	1.55	0.42	0.68	0.23	2.06	0.50
Signature	High risk vs. low risk	2.54	1.88	3.44	<0.001	2.00	1.08	3.69	0.03
**The GSE65904 dataset**									
Age	>58 vs. ≤58	1.61	0.91	2.85	0.10	1.52	1.00	2.29	0.05
Gender	Male vs. female	2.56	1.36	4.85	<0.001	0.95	0.62	1.45	0.82
Signature	High risk vs. low risk	1.89	1.11	3.23	0.02	3.55	2.25	5.58	< 0.001

### The Comparison of the Performance in Survival Prediction Between the Four-mRNA Signature With Melanoma Clark Level and Tumor Stage

**FIGURE 4:**
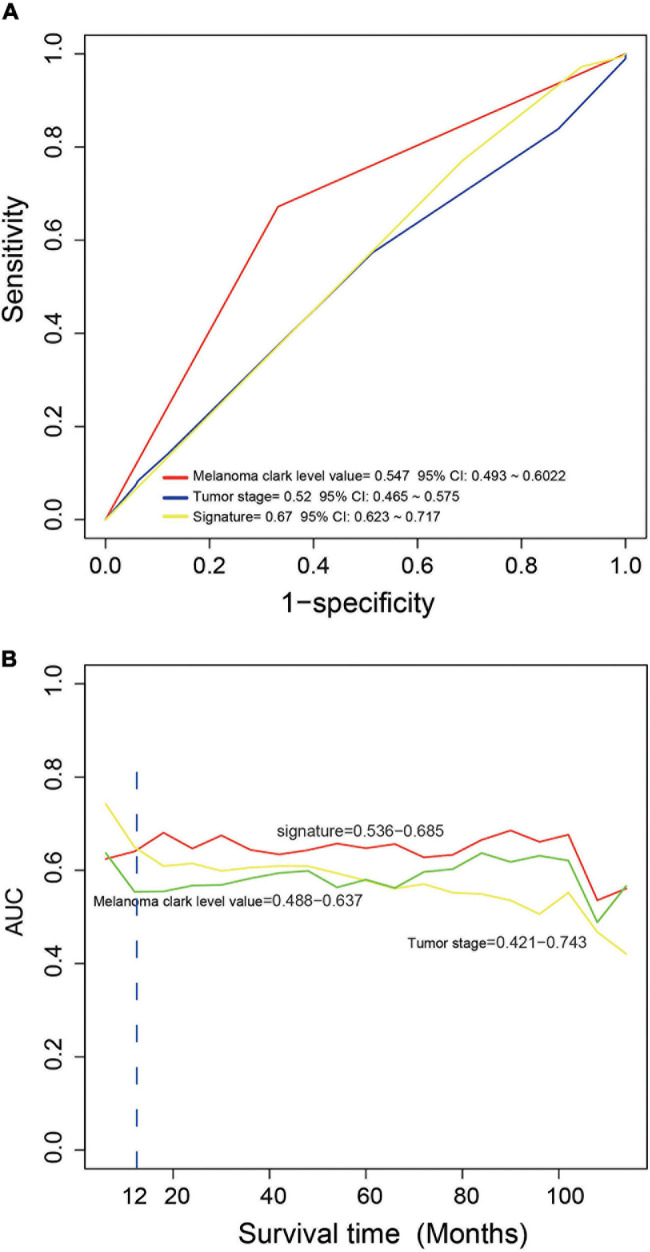
The comparison of the performance in survival prediction between the four-messenger RNA (four-mRNA) signature with tumor stage and Clark level. **(A)** ROC analysis was performed to compare the performance of the four-mRNA signature with that of tumor stage and Clark level. **(B)** TimeROC analysis was conduct to compare the performance of the four-mRNA signature with that of tumor stage and Clark level.

We compared the performance of the four-mRNA signature with other clinical prognostic markers (including melanoma Clark level and tumor stage) in predicting survival. Figure 4A shows that the four-mRNA signature was better than other clinical variables in survival prediction of the entire TCGA set (AUC signature = 0.67 vs. AUCtumor stage = 0.52 vs. AUCmelanoma Clark level = 0.55). TimeROC analysis found that the AUC values of the four-mRNA signature within 1–12 years were greater than that of the Clark level or tumor stage (Figure 4B). All these suggest that the four-mRNA signature have better performance in survival prediction of CM.

### Function Enrichment Analysis of the Four Selected mRNAs

**FIGURE 5:**
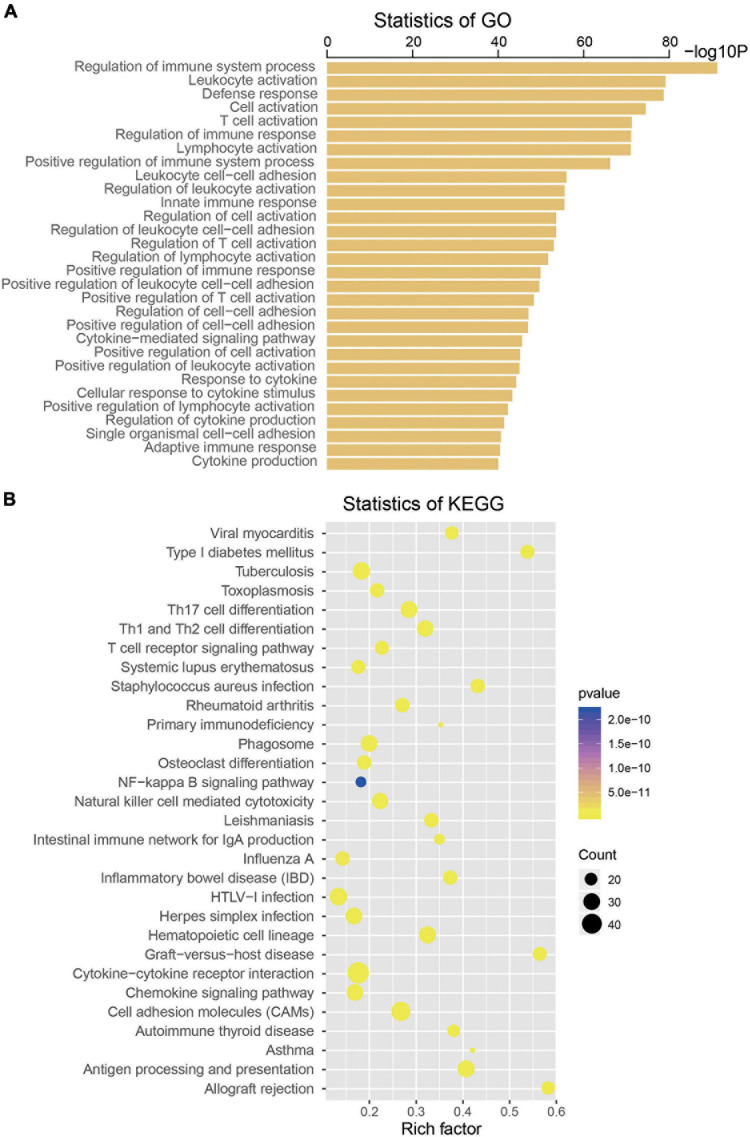
Functional enrichment analysis of the four messenger RNAs (mRNAs) in the signature by Gene Ontology **(A)** and Kyoto Encyclopedia of Genes and Genomes **(B)**.

To explore the biological roles of the four selected mRNAs from the signature in the CM, we conducted Pearson correlation test and obtained a total of 533 coexpressed genes in the TCGA dataset (coefficient > 0.5/ < -0.5, *p* < 0.001). Then, KEGG and GO analysis were performed on the above 533 coexpressed genes. We found that these coexpressed genes were significantly enriched in 739 GO terms and 45 KEGG pathways (*p* < 0.05), such as regulation of immune system process, leukocyte activation, T-cell activation, Toll-like receptor signaling pathway, Jak-STAT signaling pathway, nuclear factor (NF)-kappa B signaling pathway, suggesting the four mRNAs may influence the immune function of CM patients (top 30 are shown, Figure 5).

## Discussion

Cutaneous melanoma is a highly malignant disease with large difference in prognosis, lacking effective biomarkers for accurate survival prediction or reliable prognostic indicators. The application of precision medicine in the field of oncology highlights the prediction of individual prognosis based on gene expression profiles. Through analyzing gene expression profiles, gene expression signatures have been used to predict the prognosis of patients in different types of cancer (Burska et al., 2014), such as glioblastoma, esophageal squamous cell carcinoma, breast cancer, lung cancer, hepatocellular carcinoma, and bladder carcinoma. Therefore, using statistics and machine learning approaches, we analyzed the clinical survival information and mRNA expression of 385 melanoma patients and developed the four-mRNA signature, which could be a good prognostic biomarker for patients with CM.

After performing a variety of bioinformatics analysis methods in the TCGA group, a prognostic risk model based on the expression of four mRNAs was established to distinguish CM patients with different prognosis. This model has two advantages in predicting the prognosis of CM patients: first, it is an independent prognostic biomarker, which means that known clinical predicators such as melanoma Clark level and tumor grade will not affect its prognosis prediction. Second, the AUC value of the four-mRNA signature is greater than that of melanoma Clark and tumor grade, indicating that the four-mRNA signature has the best survival predictive performance.

The high expression of CD276 and UQCRFS1 in the four-mRNA signature was associated with short OS (Cox regression coefficient > 0), suggesting that they were risk factors for prognostic. Meanwhile, the high expression of HAPLN3 and PIP4P1 were associated with long OS (Cox regression coefficient < 0), which indicates that these two genes were beneficial factors for prognosis. Among the candidate genes, CD276 (B7-H3) is an important component of the B7 family, which can provide stimulus or inhibitory signals to enhance or weaken T-cell immune response. CD276 at the mRNA level is widely expressed in normal tissues. In tumors, CD276 or B7-H3 is reported to be highly expressed in tumors of various tissue types including melanoma, which is closely related to the poor clinical outcome of tumor patients. Studies have shown that B7-H3 plays an important role in tumor immune escape and can also affect tumor proliferation, invasion, and migration (Castellanos et al., 2017; Dong et al., 2018). In accordance with the results of this article, researchers found that B7-H3 is a significant factor in tumor progression and poor prognosis for CM patients (Tekle et al., 2012; Wang et al., 2013). HAPLN3 is a member of the hyaluronan and proteoglycan binding link protein gene family with a length of 2.1 kb and is widely expressed in various tissues (Spicer et al., 2003). HAPLN3 has been reported to play an important role in maintaining the stability of the extracellular matrix, thereby regulating the mobility and migration of tumor cells. Kuo et al. (2010) found that HAPLN3 was significantly overexpressed in breast cancer tissues, but there was no correlation between HAPLN3 gene expression and overall survival. Ubiquinol cytochrome c reductase (UQCRFS1, also named as Rieske Fe–S protein) is a catalytic submit of complex III and plays an important role in the mitochondrial respiratory chain. Researchers have found that UQCRFS1 is overexpressed in ovarian carcinoma and gastric cancer and may promote tumor development (Kaneko et al., 2003; Jun et al., 2012). Phosphatidylinositol-4, 5-bisphosphate 4-phosphatase (PIP4P1, known as TMEM55B) is an enzyme that influences cholesterol homeostasis (Medina et al., 2014) and regulates lysosomal positioning (Willett et al., 2017). However, there is no report about the role of TMEM55B in tumors.

In recent years, the role of immunity and inflammation in tumor progression has been gradually discovered (Coussens and Werb, 2002; Keibel et al., 2009). To explore the function of genes in the four-mRNA signature, GO and KEGG analyses were performed and identified that these genes were enriched in several immune and inflammation-related pathways, such as regulation of immune system process, regulation of inflammatory response and Toll-like receptor signaling pathway, Jak-STAT signaling pathway, and NF-kappa B signaling pathway, which suggests that the four-mRNA signature might influence the survival of patients with CM through regulating immune and inflammation-related pathways (Pansky et al., 2000; Janostiak et al., 2017; Luo et al., 2018; Rathore et al., 2019).

In summary, our study developed a prognostic four-mRNA signature (CD276, HAPLN3, PIP4P1, UQCRFS1) for CM, which can predict the clinical outcome of patients. Since its prognostic ability is better than the current markers (Clark level or tumor stage), the four-mRNA signature would have stronger clinical application value.

## Data Availability Statement

Publicly available datasets analyzed in this study can be found here: https://xenabrowser.net/datapages/?cohort=TCGA%20Melanoma%20(SKCM)&removeHub=https%3A%2F%2Fxena.treehouse.gi.ucsc.edu%3A443; https://www.ncbi.nlm.nih.gov/geo/query/acc.cgi?acc=GSE65904.

## Author Contributions

JL designed the study. HB and YW performed all analyses. HL checked the final manuscript. All authors reviewed the manuscript and approved the manuscript for publication.

## Conflict of Interest

The authors declare that the research was conducted in the absence of any commercial or financial relationships that could be construed as a potential conflict of interest.

## Footnotes

1 http://cancergenome.nih.gov/

2 https://xenabrowser.net/datapages/
